# Supplementary material for: Identification of photoperiod-induced specific miRNAs in the adrenal glands of Sunite sheep (Ovis aries)
Source: Front Vet Sci. 2022 Jul 22;9:888207. doi: 10.3389/fvets.2022.888207 (PMC9354845; doi:10.3389/fvets.2022.888207)
Supplement: Supplementary file 1 [file Data_Sheet_1.ZIP › Supplementary file/miRDeep2 file.zip!/miRDeep2 file/Know_Structure/SPLP42A3/oar-mir-107.pdf]

miRBase precursor	: oar-mir-107
Total read count	: 2625
oar-miR-107 read count	: 2625
remaining reads	: 0

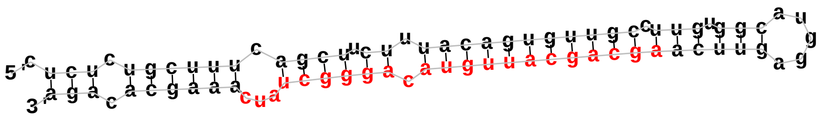

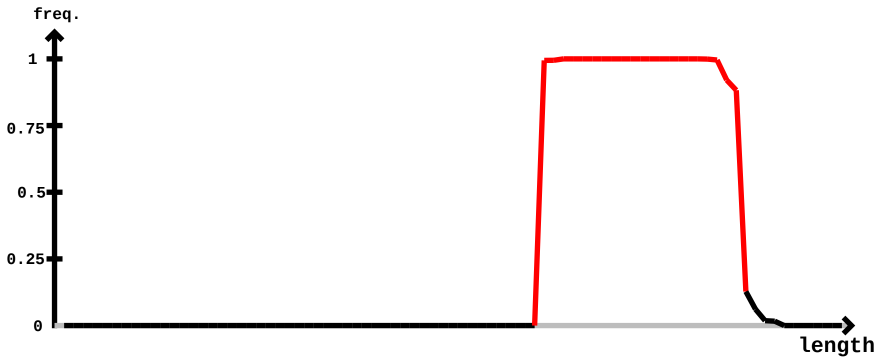


```
                                                 oar-miR-107
5'- cucucugcuuucagcuucuuuacaguguugccuuguggcauggaguucaagcagcauuguacagggcuaucaaagcacaga   -3'    exp
    .(((.((((((.((((.((.(((((((((((.(((.(((.....))))))))))))))))))).))))))...)))))).)))    reads      mm         sample
    .................................................agcagcauuguacaggg...............    2          0          seq
    .................................................agcagcauuguacagggc..............    9          0          seq
    .................................................agcagcauuguacagggcu.............    197        0          seq
    .................................................agcagcauuguacagggcua............    101        0          seq
    .................................................agcagcauuguacagggcuau...........    1976       0          seq
    .................................................agcagcauuguacagggcuauc..........    173        0          seq
    .................................................agcagcauuguacagggcuauca.........    113        0          seq
    .................................................agcagcauuguacagggcuaucaa........    5          0          seq
    .................................................agcagcauuguacagggcuauGaaa.......    34         1          seq
    ..................................................gcagcauuguacagggcuau...........    1          0          seq
    ...................................................cagcauuguacagggcuau...........    4          0          seq
    ...................................................cagcauuguacagggcuauc..........    1          0          seq
    ...................................................cagcauuguacagggcuauGaaa.......    9          1          seq
```
